# Lineage BA.2 dominated the Omicron SARS-CoV-2 epidemic wave in the Philippines

**DOI:** 10.1093/ve/veac078

**Published:** 2022-08-19

**Authors:** Yao-Tsun Li, Francisco Gerardo M Polotan, Gerald Ivan S Sotelo, Anne Pauline A Alpino, Ardiane Ysabelle M Dolor, Ma. Angelica A Tujan, Ma. Ricci R Gomez, Othoniel Jan T Onza, Angela Kae T Chang, Criselda T Bautista, June C Carandang, Maria Sofia L Yangzon, Elcid Aaron R Pangilinan, Renato Jacinto Mantaring, Alyssa Joyce E Telles, John Michael C Egana, Joshua Jose S Endozo, Rianna Patricia S Cruz, Francis A Tablizo, Jan Michael C Yap, Benedict A Maralit, Marc Edsel C Ayes, Eva Marie C de la Paz, Cynthia P Saloma, Dodge R Lim, Lei Lanna M Dancel, Mayan Uy-Lumandas, Inez Andrea P Medado, Timothy John R Dizon, Katie Hampson, Simon Daldry, Joseph Hughes, Kirstyn Brunker

**Affiliations:** Institute of Biodiversity, Animal Health and Comparative Medicine, University of Glasgow, Glasgow G12 8QQ, UK; Research Institute for Tropical Medicine, 9002 Research Drive, Filinvest Corporate City, Alabang, Muntinlupa, Metro Manila 1781, Philippines; Research Institute for Tropical Medicine, 9002 Research Drive, Filinvest Corporate City, Alabang, Muntinlupa, Metro Manila 1781, Philippines; Research Institute for Tropical Medicine, 9002 Research Drive, Filinvest Corporate City, Alabang, Muntinlupa, Metro Manila 1781, Philippines; Research Institute for Tropical Medicine, 9002 Research Drive, Filinvest Corporate City, Alabang, Muntinlupa, Metro Manila 1781, Philippines; Research Institute for Tropical Medicine, 9002 Research Drive, Filinvest Corporate City, Alabang, Muntinlupa, Metro Manila 1781, Philippines; Research Institute for Tropical Medicine, 9002 Research Drive, Filinvest Corporate City, Alabang, Muntinlupa, Metro Manila 1781, Philippines; Research Institute for Tropical Medicine, 9002 Research Drive, Filinvest Corporate City, Alabang, Muntinlupa, Metro Manila 1781, Philippines; Research Institute for Tropical Medicine, 9002 Research Drive, Filinvest Corporate City, Alabang, Muntinlupa, Metro Manila 1781, Philippines; Research Institute for Tropical Medicine, 9002 Research Drive, Filinvest Corporate City, Alabang, Muntinlupa, Metro Manila 1781, Philippines; Research Institute for Tropical Medicine, 9002 Research Drive, Filinvest Corporate City, Alabang, Muntinlupa, Metro Manila 1781, Philippines; Philippine Genome Center, National Science Complex, U.P. Campus, University of the Philippines, A. Ma. Regidor Street, Quezon City, Metro Manila 1101, Philippines; Philippine Genome Center, National Science Complex, U.P. Campus, University of the Philippines, A. Ma. Regidor Street, Quezon City, Metro Manila 1101, Philippines; Philippine Genome Center, National Science Complex, U.P. Campus, University of the Philippines, A. Ma. Regidor Street, Quezon City, Metro Manila 1101, Philippines; Philippine Genome Center, National Science Complex, U.P. Campus, University of the Philippines, A. Ma. Regidor Street, Quezon City, Metro Manila 1101, Philippines; Philippine Genome Center, National Science Complex, U.P. Campus, University of the Philippines, A. Ma. Regidor Street, Quezon City, Metro Manila 1101, Philippines; Philippine Genome Center, National Science Complex, U.P. Campus, University of the Philippines, A. Ma. Regidor Street, Quezon City, Metro Manila 1101, Philippines; Philippine Genome Center, National Science Complex, U.P. Campus, University of the Philippines, A. Ma. Regidor Street, Quezon City, Metro Manila 1101, Philippines; Philippine Genome Center, National Science Complex, U.P. Campus, University of the Philippines, A. Ma. Regidor Street, Quezon City, Metro Manila 1101, Philippines; Philippine Genome Center, National Science Complex, U.P. Campus, University of the Philippines, A. Ma. Regidor Street, Quezon City, Metro Manila 1101, Philippines; Philippine Genome Center, National Science Complex, U.P. Campus, University of the Philippines, A. Ma. Regidor Street, Quezon City, Metro Manila 1101, Philippines; Philippine Genome Center, National Science Complex, U.P. Campus, University of the Philippines, A. Ma. Regidor Street, Quezon City, Metro Manila 1101, Philippines; Philippine Genome Center, National Science Complex, U.P. Campus, University of the Philippines, A. Ma. Regidor Street, Quezon City, Metro Manila 1101, Philippines; National Institutes of Health, University of the Philippines Manila, 623 Pedro Gil Street, Ermita, Manila 1000, Philippine; Philippine Genome Center, National Science Complex, U.P. Campus, University of the Philippines, A. Ma. Regidor Street, Quezon City, Metro Manila 1101, Philippines; Research Institute for Tropical Medicine, 9002 Research Drive, Filinvest Corporate City, Alabang, Muntinlupa, Metro Manila 1781, Philippines; Research Institute for Tropical Medicine, 9002 Research Drive, Filinvest Corporate City, Alabang, Muntinlupa, Metro Manila 1781, Philippines; Research Institute for Tropical Medicine, 9002 Research Drive, Filinvest Corporate City, Alabang, Muntinlupa, Metro Manila 1781, Philippines; Research Institute for Tropical Medicine, 9002 Research Drive, Filinvest Corporate City, Alabang, Muntinlupa, Metro Manila 1781, Philippines; Research Institute for Tropical Medicine, 9002 Research Drive, Filinvest Corporate City, Alabang, Muntinlupa, Metro Manila 1781, Philippines; Institute of Biodiversity, Animal Health and Comparative Medicine, University of Glasgow, Glasgow G12 8QQ, UK; Institute of Biodiversity, Animal Health and Comparative Medicine, University of Glasgow, Glasgow G12 8QQ, UK; MRC-University of Glasgow Centre for Virus Research, Glasgow, UK; Institute of Biodiversity, Animal Health and Comparative Medicine, University of Glasgow, Glasgow G12 8QQ, UK

**Keywords:** SARS-CoV-2, genomic epidemiology, Philippines

## Abstract

The Omicron severe acute respiratory syndrome coronavirus 2 (SARS-CoV-2) variant led to a dramatic global epidemic wave following detection in South Africa in November 2021. The BA.1 Omicron lineage was dominant and responsible for most SARS-CoV-2 outbreaks in countries around the world during December 2021–January 2022, while other Omicron lineages, including BA.2, accounted for the minority of global isolates. Here, we describe the Omicron wave in the Philippines by analysing genomic data. Our results identify the presence of both BA.1 and BA.2 lineages in the Philippines in December 2021, before cases surged in January 2022. We infer that only the BA.2 lineage underwent sustained transmission in the country, with an estimated emergence around 18 November 2021 (95 per cent highest posterior density: 6–28 November), while despite multiple introductions, BA.1 transmission remained limited. These results suggest that the Philippines was one of the earliest areas affected by BA.2 and reiterate the importance of whole genome sequencing for monitoring outbreaks.

## Introduction

The continuous transmission of severe acute respiratory syndrome coronavirus 2 (SARS-CoV-2), the aetiology of the coronavirus disease (COVID-19), has led to new viral variants with accumulated genetic mutations (Rambaut et al. 2020; Harvey et al. 2021). The Omicron variant was designated by the World Health Organization (WHO) as a variant of concern (VOC) in November 2021, following previously designated Alpha, Beta, Gamma, and Delta VOCs (WHO 2021a). These SARS-CoV-2 variants each possess distinct combinations of mutations in the viral genome, particularly in the S gene, demonstrating the potential for increased transmissibility or disease severity compared with viruses isolated early in the pandemic (Dhar et al. 2021; Volz et al. 2021; Viana et al. 2022). VOCs may circulate efficiently in the population by evading antibodies derived from vaccination or prior exposures or if they elude diagnostic methods (Dhar et al. 2021; Volz et al. 2021; Lihong et al. 2022; Schmidt et al. 2022). Thus, effectively tracking the emergence and evolution of SARS-COV-2 lineages is essential to controlling the disease.

The Omicron variant was first reported in South Africa in October 2021 (Viana et al. 2022), with three divergent lineages identified (BA.1, BA.2, and BA.3). Of the three lineages, the BA.1 lineage (including its descending sub-lineages BA.1.*) has rapidly spread to dominate globally, leading to another epidemic wave during the 2021 winter (Hodcroft 2021; WHO 2021b; Chen et al. 2022). In contrast, the BA.2 and BA.3 lineages had only accounted for a minority of viral isolates by the end of 2021 (Hodcroft 2021; Chen et al. 2022). The BA.2 viruses, although phylogenetically clustered with BA.1 compared to other variants (Viana et al. 2022; Yamasoba et al. 2022), differ by at least thirty amino acids relative to BA.1 viruses (Majumdar and Sarkar 2022; Tsueng et al. 2022). Recent studies provide hints that significant genetic divergence of the two lineages results in a different replication capacity and transmissibility (Lyngse et al. 2022; UKHSA 2022; Yamasoba et al. 2022).

The Philippines was among a few countries, including Denmark and India, where the BA.2 lineage accounted for noticeable genomic data during the 2021 winter, contrary to most geographical areas mainly affected by the BA.1 lineage (Hodcroft 2021; Chen et al. 2022). The country also experienced a sharp increase in case numbers in January 2022, parallel to the global Omicron wave (Department of Health, Philippines); nevertheless, lineages contributing to local transmission and their dynamic interactions were unknown. In this study, we show using phylogenetic approaches that the BA.2 lineage but not the BA.1 lineage caused the case surge in the Philippines during the global Omicron wave. We also inferred that BA.2 circulation in the Philippines could have occurred as early as November 2021, within weeks of the lineage first being identified in South Africa. These results provide insights into how new SARS-CoV-2 lineages emerge and establish sustained transmission.

## Results

**Figure 1.**
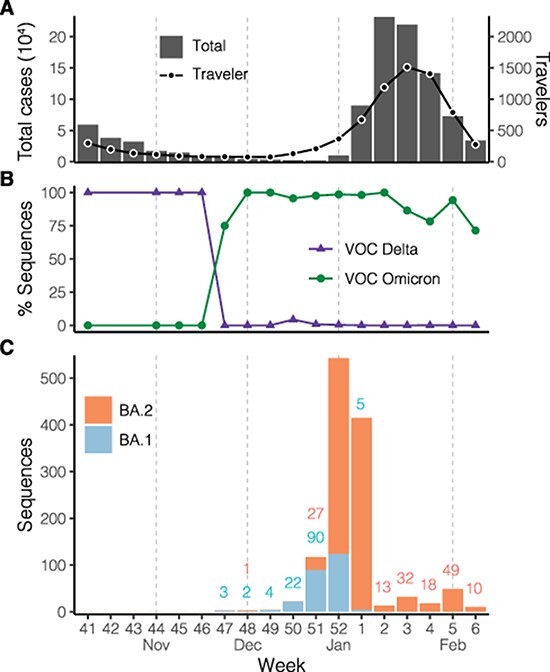
SARS-CoV-2 infections during the Omicron variant epidemic wave in the Philippines. (A) Total numbers of cases reported (bar chart) in the country and numbers of cases identified from ROFs (line) based on the case information data from the Department of Health, Philippines. (B) Proportions of variant sequences among available SARS-CoV-2 sequences as of 15 February 2022. The classification was based on the software Pangolin version 2022-02-02. (C) Numbers of available Omicron lineage sequences as of 15 February 2022. Numbers below 100 are annotated (coloured by lineage) above the bars. The BA.1 and BA.2 categories here contain the descending lineages assigned by Pangolin, e.g. BA.1 includes the BA.1.1 lineage. *X*-axis labels indicate the epidemiological week defined by the US-CDC (Centers for Disease Control), which corresponds to the ISO (International Organization for Standardization) week starting on Sunday.

The epidemic wave associated with the Omicron variant in the Philippines started in December 2021. Based on case information available from the Department of Health, Philippines, reported COVID-19 case numbers rose towards the end of 2021, reaching a peak with over 30,000 cases per day in Week 2 (10–16 January), 2022, before rapidly declining to fewer than 5,000 cases per day in Week 6 (7–13 February) (Fig. 1A, bar chart). Numbers of cases identified from returning overseas Filipinos (ROFs) demonstrate a remarkably similar epidemic profile to the reported domestic cases (Fig. 1A, line), suggesting a linkage of global SARS-CoV-2 transmission to the domestic epidemic.

To better understand the transmission of SARS-CoV-2 viruses leading to the case surge, we combined sequence data collected by the Genomic Epidemiology of COVID in the Philippines (GECO) project and data available on the GISAID (Shu and McCauley 2017). The sequences in the Philippines show the Delta variant, including lineages B.1.617.2 and AY.*, as being the dominant circulating variant in the country before being replaced by the Omicron variant (lineages BA.*) (Fig. 1B). Specifically, the proportion of sequenced cases belonging to the Omicron variant exceeded the Delta variant in November 2021, about 1 month before the rise of the epidemic wave (Fig. 1A and 1B), and the Omicron variant has accounted for the majority of sequences since. Among the Omicron variant viruses in the Philippines, the BA.1 lineage, first identified on 22 November, had accounted for more available sequences than its sister lineage BA.2 until the last week of 2021 (Fig. 1C), although the numbers of both lineages were low at most time points during November–December 2021. In contrast, BA.2 has been the most prevalent since the lineage drastically increased in the last week of the year 2021 (Fig. 1C).

**Figure 2.**
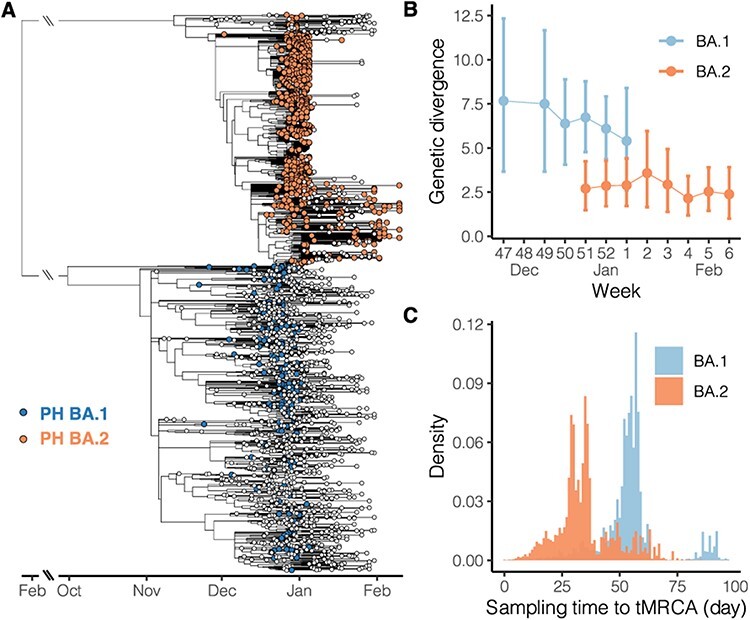
Phylogenetic relationship of SARS-CoV-2 Omicron variants isolated in the Philippines. (A) Time-scaled tree was inferred by TreeTime using Omicron variant genome sequences isolated in the Philippines and from the global database. The blue and orange tips indicate the BA.1 and BA.2 lineage viruses, respectively, isolated in the Philippines, whereas the white tips indicate the viruses isolated in the other countries. Long branches descending from the common ancestor of BA.1 and BA.2 are shortened. (B) Average genetic divergence of viruses isolated in the Philippines. Error bars represent 95 per cent bootstrap percentiles. (C) Distribution of time intervals from tMRCA to the isolation time. The time intervals were calculated based on pairs of Philippine taxa on the time-scaled tree (A); for each pair, the larger time difference was recorded.

BA.1 and BA.2 viruses isolated in the Philippines show divergent distributions on the phylogeny inferred by the whole viral genome. With global strains sampled in an unbiased manner against the proximal Philippines isolates, the BA.1 viruses isolated in the Philippines are intermixed with the non-Philippine viruses on the temporal phylogenetic tree, suggesting a large number of introductions. In contrast, BA.2 viruses are largely clustered in one clade, in which the most genetically similar virus of each Philippine isolate is nearly always from the Philippines (Fig. 2A). The estimation of introductory events by ancestral state reconstruction using the parsimony method also supports this observation: 136 potential introductory events to the Philippines were identified in the BA.1 lineage, which led to clusters with a mean sample size below 2, compared with 25 potential introductory events identified in the BA.2 lineage, which led to two major clusters with sizes of 699 and 206 in addition to the remaining clusters each having less than 10 samples.

We therefore hypothesise that the two Omicron lineages in the Philippines demonstrate different epidemiological patterns. We assume if most genomic samples were collected from the context of sustained transmission rather than sporadic introduction, genetic differences between sequences isolated in approximate time points would be minimal. Additionally, if most samples were collected from the sustained transmission, viral taxa would coalesce to close common ancestors on the phylogeny, in contrast to deeper common ancestors likely shared by taxa isolated in unlinked transmission chains. Our results show that BA.1 lineage sequences grouped by week have greater average genetic differences compared with BA.2 lineage sequences (Fig. 2B). Especially, among the 2 or 3 weeks where both lineages are available (week 51-52), average nucleotide differences shared by paired BA.1 sequences are more than twice that of the BA.2 sequences. Although the BA.2 lineage in the Philippines has more overall samples than the BA.1 lineage (Fig. 1C), weekly genetic differences of BA.2 remain stable throughout the studied intervals, averaging about 2.5 nucleotide differences among sequences isolated each week (Fig. 2B). Furthermore, when pairwise time intervals between the most recent common ancestor and isolation time are compared between the two lineages, the BA.1 lineage shows greater intervals than the BA.2 lineage (Fig. 2C). The average time intervals of the BA.1 and BA.2 lineages are 54 and 35 days, respectively, indicating that each pair of BA.1 viruses have deeper common ancestors than those of the BA.2 viruses. Combined with the observations where no distinguishable Philippines clade was formed among the BA.1 global isolates (Fig. 2A), these comparative analyses suggest that the BA.2 but not BA.1 lineage underwent sustained transmission in the Philippines.

**Figure 3.**
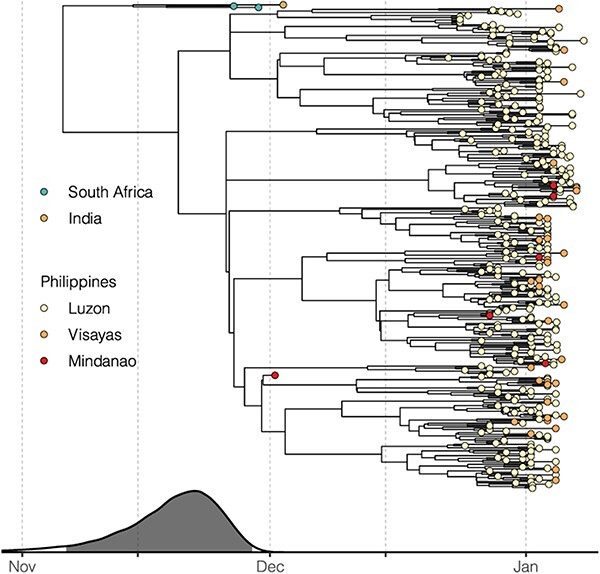
Introduction of the BA.2 lineage in the Philippines. The time-scaled tree was inferred by BEAST using BA.2 genomes isolated in the Philippines along with the genomes of early global BA.2 viruses. The estimated tMRCAs with 95 per cent HPD illustrated by the grey area are aligned with the phylogenetic tree. Tips are coloured according to the location of isolation. Red shades for viruses isolated in the Philippines indicate the three island groups in the country.

To gain more insights into the introduction of the BA.2 lineage in the Philippines, we estimated the time of the most recent common ancestor (tMRCA) based on the BA.2 genomic sequences isolated in the country in addition to early BA.2 strains identified globally (Fig. 3). The estimated tMRCA is 18 November 2021 (95 per cent highest posterior density (HPD), 6–28 November), 2 weeks before the first BA.2 case identified in the Philippines. The estimated tMRCA does not significantly differ from the root of the BA.2 temporal phylogeny (95 HPD, 23 October–17 November), which may corroborate with the previous understanding that the Philippines was one of the earliest countries where the circulation of the BA.2 lineage was discovered. No apparent diffusion pattern was observed based on the geographical distribution of the phylogeny (Fig. 3). Viruses isolated from the three major island groups are generally mixed on the subclades, providing evidence of extensive domestic transmission and underlying circulation before increased sampling in January. Indeed, available sequences assigned as BA.1 have only been isolated from nine administrative regions, compared with BA.2 isolated from sixteen regions (Supplementary Figure). Among these regions, early BA.2 from the National Capital Region (Luzon island group), Ilocos (Luzon), and the Eastern Visayas (Visayas) show statistically significant clustering on the phylogeny (Supplementary Table).

## Discussion

In this study, we show that the BA.2 but not the BA.1 lineage of the Omicron variant fits the scenario of community transmission in the Philippines based on viral genomic data. With the majority of sequences isolated during the country’s latest case rise identified as the BA.2 lineage, we propose that the Omicron epidemic wave in the Philippines was mostly driven by BA.2 viruses in contrast to most other Omicron waves seen during this time globally. In most countries with continuous genomic surveillance, including South Africa, the UK, and the USA, case peaks associated with the Omicron variant during the 2021 winter were caused by the BA.1 (including descendant BA.1.*) lineage (Chen et al. 2022; Tsueng et al. 2022). In contrast, the BA.2 lineage was only observed to be dominant in a few countries besides the Philippines, including India, Nepal, Bangladesh, Denmark, and Qatar, by the end of January 2022 based on the available genomic data (Hodcroft 2021; Chen et al. 2022). Our results in the Philippines thus present a special case whereby the BA.2 lineage led to the local transmission without a previous extensive BA.1 outbreak.

Since March 2022, there have been clear signs that BA.2 has replaced BA.1 in several geographical regions (Chen et al. 2022; Tsueng et al. 2022). Understanding how the BA.2 lineage became dominant over the previous circulating variants in the Philippines could provide important insights for controlling BA.2 in affected countries. As there appears to have been only a low level of local BA.1 circulation in the Philippines, it is not directly clear whether virological properties of the two Omicron lineages, including intrinsic transmissibility or antigenicity (Lyngse et al. 2022; Schmidt et al. 2022; UKHSA 2022; Yamasoba et al. 2022), competitively determined the epidemic outcome through the selection of a more fit strain. Since Omicron emerged, routine testing using the S-gene target failure marker has been implemented to detect and curtail the spread of BA.1 by distinguishing it from the Delta variant. However, this method identifies BA.1 but rarely BA.2 based on the deletions in the viral S gene (Majumdar and Sarkar 2022; UKHSA 2022) and therefore may have led to reduced detection of BA.2, potentially favouring the emergence and spread of BA.2 viruses. This point emphasises the importance of whole genome sequencing as part of SARS-CoV-2 surveillance programmes.

We estimated the most recent common ancestor of the Philippine BA.2 viruses to be in late November 2021, about 2 weeks before the first detected case of BA.2 in the Philippines. This estimate by Bayesian phylogenetic reconstruction is corroborated with the time-scaled tree inferred using the maximum likelihood (ML) method (Fig. 2A, the tMRCA of the major clade formed by Philippine taxa), suggesting that local spread could have started in November. The BA.2 lineage was first described in South Africa in early November 2021, along with other Omicron lineages (Viana et al. 2022). Local BA.2 transmission was then reported in Denmark where the first BA.2 case was identified on 5 December 2021 (Fonager et al. 2022), and the country accounted for most global BA.2 sequences as of mid-January 2022 (>5,000) based on GISAID. India was also identified as one of the earliest BA.2-affected countries, with BA.2 sequences isolated as early as November 2021 (Hodcroft 2021). The temporal phylogeny estimated from our BA.2 data set ascertained multiple introductory events, based on the topology of the tree and limited sampling in early December (Fig. 3). Despite uncertainty in the exact origin of currently circulating BA.2 viruses in the Philippines, the estimated date of emergence appears robust to sampling effects. The two BA.2 subclades, either with or without the first isolate on 3 December, could still trace the most recent common ancestor’s origin before December (Figs 2A and 3).

The estimated BA.2 emergence time coincides with the general de-escalation of control measures in the Philippines. The de-escalation may be attributed to the decreasing number of identified COVID-19 infections in November 2021. At this time, new COVID-19 infections reached their lowest number in the previous 11 months (Department of Health, Philippines). There was also general optimism and anticipation for the Christmas season, during which migrant Filipino workers come home to celebrate with their families. How migrant workers contributed to local transmission warrants further research.

Variation in sequencing rates across administrative regions in the Philippines renders our genomic data unlikely to reflect the domestic geographical diffusion of lineages in the Philippines. Our comparative analyses between BA.1 and BA.2 lineages, nevertheless, are less affected by undersampling since the analyses were based on the topology of the phylogeny, and the sample selection for sequencing would not be biased by lineage. Importantly, routine genomic surveillance in the Philippines shows no evidence of emergence of the BA.1 lineage (https://geco-ph.github.io/GECO-covid). Retrospective sequencing of Philippine and global samples will facilitate an improved understanding of the BA.2 origin and reconstruction of viral diffusion dynamics during the pandemic. The root of the temporal phylogeny of BA.2 is estimated on 5 November, which is very close to the emergence date estimated by a recent study (6 November) (Yamasoba et al. 2022).

In summary, we show that the epidemic wave in the Philippines was driven by the BA.2 Omicron lineage but not BA.1, although both lineages were sampled before and during the rise in case numbers. Also, the BA.2 viruses causing the country’s epidemic circulated in the Philippines before December 2021, in parallel to Denmark as one of the earliest countries where local BA.2 outbreaks occurred outside of Southern Africa. Our study highlights the value of phylogenetic methods for understanding viral transmission and the need to rapidly generate genomic data to inform control strategies.

## Materials and methods

### Genomic surveillance in the Philippines

SARS-CoV-2 sequences were collected under the framework of a collaborative project, GECO. The project aims to use viral genomes generated by nanopore sequencing to inform public health measures against COVID-19. SARS-CoV-2 PCR-positive RNA samples from partnered sub-national laboratories were subjected to whole genome sequencing using the ARTIC network multiplex PCR workflow performed at a national core laboratory, the Research Institute for Tropical Medicine. The ARTIC network bioinformatic pipeline was used to generate consensus sequences from raw output files with steps of basecalling, de-multiplexing, mapping, and polishing (https://artic.network/ncov-2019). As of 15 February 2022, 1,055 consensus sequences of SARS-CoV-2 have been generated by the project.

### Sequence data preparation

To compile all available genomic data from the Philippines and fit the domestic isolates in the context of global virus transmission, all SARS-CoV-2 sequences and metadata were downloaded from GISAID on 15 February 2022 (EpiCoV, https://www.gisaid.org) (Shu and McCauley 2017). The downloaded data were first split into Philippine/non-Philippine portions based on the location of isolation, in which the Philippine data deposited in GISAID were then combined with data collected by the GECO project. An Omicron data set containing all Omicron sub-lineages from the Philippines and a BA.2 lineage data set containing only BA.2 lineage data from the country were prepared according to the Pango lineages (Rambaut et al. 2020) assigned to each sequence by Pangolin version 2022-02-02 (https://github.com/cov-lineages/pangolin) or information provided by GISAID. For each data set, 1,500 global proximal strains genetically similar to the Philippine strains were sampled by the Nextstrain bioinformatic pipeline (Hadfield et al. 2018). The quality of the compiled genomic data was evaluated by Nextclade CLI v1.10.3 (Aksamentov et al. 2021). We filtered out sequences that had more than five private mutations or an SNP cluster. Sequences shorter than 27,000 nucleotides or sequences excluded by Nextclade due to too many ambiguous sites were also removed from the data sets. The accession numbers of the sequences analysed in this study have been compiled as an EPI SET (EPI_SET_20220430vo).

### Phylogenetic and other genetic analyses

Curated whole genome SARS-CoV-2 sequences were aligned using Nextalign v1.9 (Aksamentov et al. 2021), and the alignments supplemented with a reference strain Wuhan/Hu-1/2019 were subject to ML tree inference using IQ-TREE v2.2.0 (Minh et al. 2020). To focus on domestic transmission in the Philippines and cross-border events, we subsampled the compiled Omicron data set by selecting a taxon in each monophyletic group that comprised only strains isolated from the same country outside the Philippines, and the reduced data set was then used to rebuild another ML tree. Based on the resulting ML tree, the time-scaled tree of the Omicron variant was estimated using TreeTime v0.8.5 with a clock rate of 0.0008. The tMRCAs of specific taxa can be parsed from the internal nodes in the time-scaled tree.

To more closely explore the timing of the BA.2 introduction, a Bayesian phylogenetic framework was implemented with BA.2 sequences collected in the Philippines. A more strictly filtered BA.2 data set was prepared with sequences annotated as good quality by Nextclade. With this, BA.2 genome data for the time-calibrated phylogeny subsequently included all filtered Philippine BA.2 sequences isolated before 15 January 2022, with apparently divergent BA.2 strains removed (*n* = 19), and early BA.2 strains in South Africa and India. A time-scaled phylogeny was inferred using BEAST v10.4 (Suchard et al. 2018) facilitated by the BEAGLE library v3.1 for better computational performance (Ayres et al. 2019). We employed an HKY plus gamma substitution model and a strict molecular clock with an exponential demographic prior in the Bayesian analyses. Markov chain Monte Carlo (MCMC) analysis was run for 100 million steps and sampled every 10,000 steps. Three parallel runs were performed and combined with a burnin of 10 million per chain using LogCombiner (Suchard et al. 2018). Parameters logged during the MCMC runs were inspected by Tracer v1.7.1 (Rambaut et al. 2018). A summarised maximum clade credibility tree was inferred using TreeAnnotator (Suchard et al. 2018).

Genetic divergence was calculated by sequence length times genetic diversity (pi) (Nei et al. 2000). Introductory events and the local clusters were identified using clusterfunk v0.1.0 (https://github.com/snake-flu/clusterfunk) with phylogenetic trees inferred by IQ-TREE. Statistical correlation between the locations of isolation and the phylogeny was detected by BaTS v0.9 with 1,000 posterior trees subsampled from the MCMC process (Parker, Rambaut, and Pybus 2008). All phylogenetic trees were visualised by ggtree (Yu et al. 2017).

## Supplementary Material

veac078_Supp

## Data Availability

Genomic data collected by the GECO project are available on GISAID, and the accession numbers of the sequences analysed in this study have been compiled as an EPI SET (EPI_SET_20220430vo). The XML file required for BEAST and details of genetic analyses including phylogenetic trees are available at https://github.com/GECO-PH/GECO-covid/tree/main/manuscript_BA2.

## References

[R1] Aksamentov I. et al. (2021) ‘Nextclade: Clade Assignment, Mutation Calling and Quality Control for Viral Genomes’, *Journal of Open Source Software*, 6: 3773.

[R2] Ayres D. L. et al. (2019) ‘BEAGLE 3: Improved Performance, Scaling, and Usability for a High-Performance Computing Library for Statistical Phylogenetics’, *Systematic Biology*, 68: 1052–61. 31034053 10.1093/sysbio/syz020 PMC6802572

[R3] Chen C. et al. (2022) ‘CoV-Spectrum: Analysis of Globally Shared SARS-CoV-2 Data to Identify and Characterize New Variants’, *Bioinformatics*, 38: 1735–7. 34954792 10.1093/bioinformatics/btab856 PMC8896605

[R4] Department of Health (Department of Health), Philippines . COVID-19 Tracker. <https://doh.gov.ph/covid19tracker> accessed 25 Feb 2022.

[R5] Dhar M. S. et al. (2021) ‘Genomic Characterization and Epidemiology of an Emerging SARS-CoV-2 Variant in Delhi, India’, *Science*, 374: 995–9. 34648303 10.1126/science.abj9932 PMC7612010

[R6] Fonager J. et al. (2022) ‘Molecular Epidemiology of the SARS-CoV-2 Variant Omicron BA.2 Sub-lineage in Denmark, 29 November 2021 to 2 January 2022’, *Eurosurveillance*, 27: 2200181. 10.2807/1560-7917.ES.2022.27.10.2200181 PMC8915403 35272746

[R7] Hadfield J. et al. (2018) ‘Nextstrain: Real-time Tracking of Pathogen Evolution’, *Bioinformatics*, 34: 4121–3. 29790939 10.1093/bioinformatics/bty407 PMC6247931

[R8] Harvey W. T. et al. (2021) ‘SARS-CoV-2 Variants, Spike Mutations and Immune Escape’, *Nature Reviews Microbiology*, 19: 409–24. 34075212 10.1038/s41579-021-00573-0 PMC8167834

[R9] Hodcroft E. (2021) CoVariants: SARS-CoV-2 Mutations and Variants of Interest. <https://covariants.org/> accessed 30 Apr 2022.

[R10] Lihong L. et al. (2022) ‘Striking Antibody Evasion Manifested by the Omicron Variant of SARS-CoV-2’, *Nature*, 602: 676–81. 35016198 10.1038/s41586-021-04388-0

[R11] Lyngse F. P. et al. (2022) ‘Transmission of SARS-CoV-2 Omicron VOC Subvariants BA.1 and BA.2: Evidence from Danish Households’, *medRxiv*. 2022.01.28.22270044.

[R12] Majumdar S. , and Sarkar R. (2022) ‘Mutational and Phylogenetic Analyses of the Two Lineages of the Omicron Variant’, *Journal of Medical Virology*, 94: 1777–9. 34964502 10.1002/jmv.27558 PMC9015627

[R13] Minh B. Q. et al. (2020) ‘IQ-TREE 2: New Models and Efficient Methods for Phylogenetic Inference in the Genomic Era’, *Molecular Biology and Evolution*, 37: 1530–4. 32011700 10.1093/molbev/msaa015 PMC7182206

[R14] Nei M , Kumar S, Nei EPP of BM. (2000) *Molecular Evolution and Phylogenetics*. USA: Oxford University Press.

[R15] Parker J. , Rambaut A., and Pybus O. G. (2008) ‘Correlating Viral Phenotypes with Phylogeny: Accounting for Phylogenetic Uncertainty’, *Infection, Genetics and Evolution*, 8: 239–46. 10.1016/j.meegid.2007.08.001 17921073

[R16] Rambaut A. et al. (2018) ‘Posterior Summarization in Bayesian Phylogenetics Using Tracer 1.7’, *Systematic Biology*, 67: 901–4. 29718447 10.1093/sysbio/syy032 PMC6101584

[R17] ——— et al. (2020) ‘A Dynamic Nomenclature Proposal for SARS-CoV-2 Lineages to Assist Genomic Epidemiology’, *Nature Microbiology*, 5: 1403–7. 10.1038/s41564-020-0770-5 PMC7610519 32669681

[R18] Schmidt F. et al. (2022) ‘Plasma Neutralization of the SARS-CoV-2 Omicron Variant’, *New England Journal of Medicine*, 386: 599–601. 35030645 10.1056/NEJMc2119641 PMC8757565

[R19] Shu Y. , and McCauley J. (2017) ‘GISAID: Global Initiative on Sharing All Influenza Data – from Vision to Reality’, *Eurosurveillance*, 22: 30494. 10.2807/1560-7917.ES.2017.22.13.30494 PMC5388101 28382917

[R20] Suchard M. A. et al. (2018) ‘Bayesian Phylogenetic and Phylodynamic Data Integration Using BEAST 1.10’, *Virus Evolution*, 4: vey016. 10.1093/ve/vey016 PMC6007674 29942656

[R21] Tsueng G. et al. (2022) Outbreak.info Research Library: A Standardized, Searchable Platform to Discover and Explore COVID-19 Resources. 2022.01.20.477133. <https://www.biorxiv.org/content/10.1101/2022.01.20.477133v3>. 10.1038/s41592-023-01770-w PMC10393269 36823331

[R22] UKHSA . (2022) SARS-CoV-2 Variants of Concern and Variants under Investigation in England Technical Briefing 39.

[R23] Viana R. et al. (2022) ‘Rapid Epidemic Expansion of the SARS-CoV-2 Omicron Variant in Southern Africa’, *Nature*, 603: 679–86. 35042229 10.1038/s41586-022-04411-y PMC8942855

[R24] Volz E. et al. (2021) ‘Assessing Transmissibility of SARS-CoV-2 Lineage B.1.1.7 in England’, *Nature*, 593: 266–9. 33767447 10.1038/s41586-021-03470-x

[R25] WHO . (2021a) Classification of Omicron (B.1.1.529): SARS-CoV-2 Variant of Concern. <https://www.who.int/news/item/26-11-2021-classification-of-omicron-(b.1.1.529)-sars-cov-2-variant-of-concern> accessed 15 Feb 2022.

[R26] ——— (2021b) Update on Omicron <https://www.who.int/news/item/28-11-2021-update-on-omicron> accessed 15 Feb 2022.

[R27] Yamasoba D. et al. (2022) ‘Virological Characteristics of the SARS-CoV-2 Omicron BA.2 Spike’, *Cell*, 85: 2103–15. 10.1016/j.cell.2022.04.035 PMC9057982 35568035

[R28] Yu G. et al. (2017) ‘Ggtree: An R Package for Visualization and Annotation of Phylogenetic Trees with Their Covariates and Other Associated Data’, *Methods in Ecology and Evolution*, 8: 28–36.

