# Supplementary material for: Lineage BA.2 dominated the Omicron SARS-CoV-2 epidemic wave in the Philippines
Source: Virus Evol. 2022 Aug 19;8(2):veac078. doi: 10.1093/ve/veac078 (PMC9452094; doi:10.1093/ve/veac078)
Supplement: veac078_Supp [file veac078_supp.zip › Supp_data.pdf]

Lineage BA.2 dominated the Omicron SARS-CoV-2 epidemic wave in the Philippines

Supplementary data

Supplementary Table. Correlation between geographical location and phylogeny.

Region	Observed mean (95% CI)	Null mean (95% CI)	Significance
**Global**	2.36 (1-3)	1.00 (1-1.02)	0.005
**National Capital Region**	7.47 (6-11)	6.15 (5.68-6.84)	0.04
Central Luzon	2.07 (2-3)	1.48 (1.28-2.01)	0.06
Cagayan Valley	1.03 (1-1)	1.04 (1-1.09)	1.0
Calabarzon	2.60 (2-4)	2.23 (2.06-2.44)	1.0
Western Visayas	1.15 (1-2)	1.17 (1.07-1.40)	1.0
Cordillera Administrative Region	1.01 (1-1)	1.01 (1.0-1.03)	1.0
**Ilocos**	2.18 (2-3)	1.11 (1.03-1.21)	0.015
Soccsksargen	1.0 (1-1)	1.00 (1.0-1.01)	1.0
**Eastern Visayas**	3.0 (3-3)	1.14 (1.05-1.33)	0.005
Davao Region	1.02 (1-1)	1.01 (1.0-1.03)	1.0
Central Visayas	1.07 (1-2)	1.03 (1.00-1.08)	1.0
Mimaropa	1.0 (1-1)	1.0 (1.0-1.0)	1.0

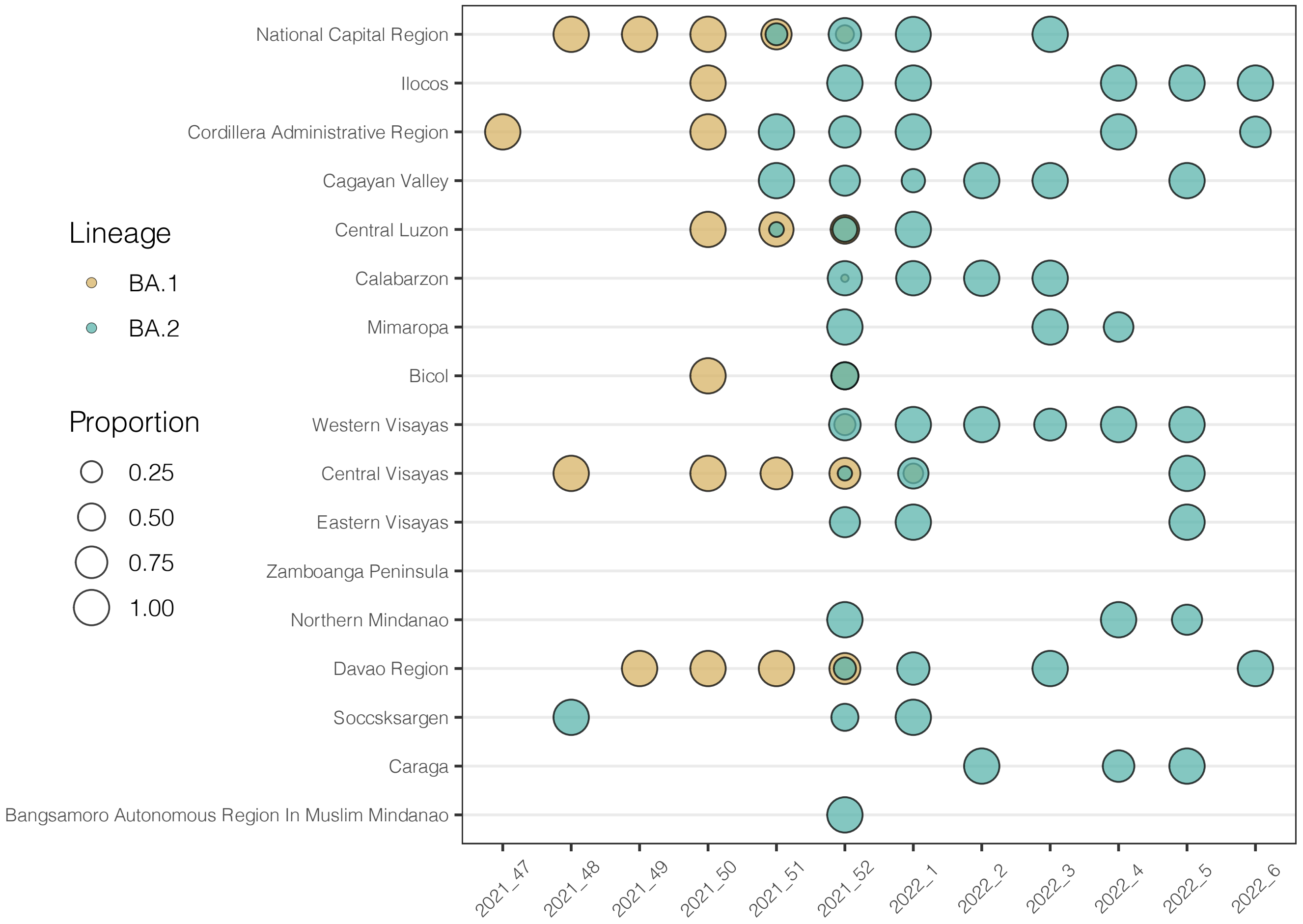

Supplemnentary Figure. Visualisation of locations of BA.1/BA.2 samples against time. X-axis labels represent year plus week and circle sizes are scaled to the proportion of the lineage per location per week. Data source is identical to Figure.1C.
